# Discussions before the Mississippi Valley Dental Association

**Published:** 1878-04

**Authors:** 


### Proceedings of Societies.

### DISCUSSIONS BEFORE THE MISSISSIPPI VALLEY DENTAL ASSOCIATION.

WEDNESDAY MORNING SESSION, MARCH 6, 1878.

The first subject announced for discussion was Dental Hygiene.

Dr. Taft opened the discussion. He said: It seems to me that every member ought to have looked over these subjects before coming here so as to be ready at once to enter upon this discussion. I regret very much that it falls to my lot generally to open the discusssion on these subjects, but I do not think we should sit waiting on each other and so allow the time to go by unoccupied.

This is a very broad subject. Such a thing as dental hygiene does not exist independent of general hygiene—that is, care for the health of the teeth will amount to little or nothing if the principles of general hygiene are neglected. It involves and embraces all the laws of health, and all things that pertain to the welfare of the animal economy. In order to do the best for the teeth, attention must be given to the entire system. Hygiene is not the treatment of disease, but the warding off of disease, or anything that would produce disease if permitted to operate.

There is a very circumscribed view taken of this subject by dentists, who think that if they are posted upon the organs of the mouth their whole duty is accomplished. Ventilation, pure air, food, clothing, temperature, all these should be thoroughly studied by the dentist, and the books written on these subjects should be his text books.

He should also subscribe for and read journals of health; and for general interest and instruction I know of none better than the Sanitarian.

Now this matter of hygiene is a subject of exceeding interest in respect to development and growth and also at and after maturity, the teeth of the mother during the periods of gestation and lactation will be influenced very much by the general conditions existing during these times. If the system is neglected in all other respects, the teeth will necessarily suffer. It has been said that attention in these directions devolves upon the general physician. That is true, but it does not relieve the dentist of his responsibility. It is true, perhaps, that there are two or three other duties more particularly devolving upon the dentist, such as operations on the teeth and what he shall do for their preservation. There is not much for the dentist to do in a manipulative way upon the teeth for their preservation. Of course he must remove deposits that accumulate from time to time. The dentist should ward off everything that would be injurious to the teeth. Often a dentist will examine the teeth of a patient and discovering two, three, four or five teeth that need filling they go to work and fill them, while salivary calculus may be accumulating in large quantities and they will entirely overlook or neglect it at least until after the filling has all been done.

The mucous membrane sometimes becomes locally affected from some cause, and this acts unfavorably upon the teeth, and where salivary calculus has formed that will affect the mucous membrane, this should be attended to first. Much can be done by the patient himself, but it is no light task to sufficiently impress upon him or her the importance of cleanliness and care, so that it shall be continued right along. It is not difficult to get them to promise, but it is difficult to get them to perform. Some dentists can succeed in this and some can not, but the subject is entirely too much overlooked by all. I think it might not be amiss to set apart an hour of our time here and examine each others teeth, and I would be very much surprised indeed if we found them all perfectly clean and free from deposits.

The President—I am under the impression that the members would have business to attend to somewhere else about that time.

Dr. Meredith thought Dr. Taft should feel complimented by having to open the discussion of this subject, but there was one unfortunate thing about it and that was, that the Doctor exhausted the subject so fully that nothing was left for any one else to say. He had often thought that if the Association could discover some method of making people take care of their teeth it would be the best thing they could do. He reported the case of a young lady whose parents could ill afford to have her teeth put in proper condition, and after putting her teeth in good condition, he told her the money and labor would all be thrown away if she did not take care of her teeth, and gave her verbal instructions and also a pamphlet containing the necessary information, and she promised faithfully to attend to them, and seeing her mother some time afterward, he learned that the young lady only cleaned her teeth once a day, in the morning, with a brush, and never drew anything between them at all, and he would not be surprised to have her come back at any time with her teeth almost as bad as ever and blame him for not doing good work.

Dr. Osmond—It is pretty hard to make every one do what is right; we don't always do it ourselves. People will readily promise to take good care of their teeth, but they seldom do it. He generally compromised with patients by getting them to clean their teeth once a day, in the evening. If people clean their mouth in the morning, they are only locking the door after the horse is stolen. He always told his patients, If you will clean your mouth for two weeks and then omit it one night, the taste you will have in your mouth next morning will drive you back to first principles. I am often asked what tooth powder is the best. This is a hard question to answer, but I prefer to have them know what they are using instead of purchasing some vile nostrum, so I generally recommended a powder consisting of two parts charcoal, two parts prepared chalk, and two parts orris root. As to Sozodont, I say don't use it. There are more teeth spoiled by that vile compound than you can tell. If you wash your hands with soap some of the cuticle is removed, but as nature is constantly reproducing the cuticle this does not matter, but with the teeth this is not true. The agents employed in cleaning the teeth should neither be acid nor alkali.

Dr. Keely—Do you consider that Sozodont has an virtue beyond the little soap there is in it?"

Dr. Osmond—I don't think it has any virtue at all.

Dr. H. A. Smith thought much was said about nostrums that was not warranted. He believed that Sozodont had been analyzed and found not to be injurious, and he generally told his patients that it was not injurious. He did not like the use of charcoal because of its sharpness, which enabled it to wear under the edge of the gums. Its only good quality was as a disinfectant and it soon lost that power. He also spoke at some length on the carelessness of patients with regard to their teeth. He said further: "I think people who contemplate marriage should have the teeth most carefully attended to, that they may be in perfect order. You know the children of drunkards are more likely to become drunkards, and I don't see why those with decayed teeth may not transmit their defects to offspring."

Dr. Berry thought the importance of keeping the gums in good condition very great. Frequently see sound teeth lost from diseases of the gums. He thought the teeth were benefited by the use of powders once or twice a week,

Dr. Keely said he had been battling against the use of charcoal for twenty years, and wanted to say for the benefit of the young members that where patients use charcoal, they almost invariably have trouble from the fine particles working down around the gums, and to remove it is as difficult as to remove salivary calculus. For children he thought nothing was better than prepared chalk. He often prescribed some simple dentrifice for the purpose only of getting people to clean their teeth.

Dr. H. M. Reid—I think there is no one thing which dentists so much neglect as the cleaning of patient's teeth. One reason is that we don't get half paid for the work done. I have known men who were good operators and would do splendid work on filling, but when cleansing teeth, don't half do the work. I recently had a case on which I spent over four hours cleansing the teeth, and I got eight dollars for it. I couldn't well charge more, but I am convinced we don't charge enough for that kind of work, and that I think is the chief reason why the work is so poorly done.

On motion the society adjourned until two o'clock p. m.

AFTERNOON SESSION.

The society assembled at two o'clock, and the discussion of the subject "Dental Hygiene" was resumed by Dr. Taft as follows:

Dr. Taft—I am very happy to hear this discussion pressed with such a degree of definiteness in the various directions which have been pursued. As to the subject of dentrifices, I think many persons do not need anything, while others require simply some slight friction to take away stains and deposits. In some instances more is required. Alkali is often a good thing, if there is any deposits or secretion of acids. Lime water or carbonate of chalk is also serviceable at times, while something else is required. If there is a condition of fœter in the mouth, then some disinfectant is called for, permanganate of potash or a little wash of salycilic acid will be found beneficial. Sometimes a stimulant is needed, then select the appropriate medicine and embody it in the dentrifice. Nothing is claimed for charcoal, but its power as a disinfectant, and the truth is that it soon becomes saturated and will no longer absorb the offensive materials, so that it is far better to use something else that will meet the difficulty better than the mere absorbing power of charcoal. Salycilic acid is both a disinfectant and a good antiseptic, so is carbolic acid, creosote and permanganate of potash." He thought Sozodont mischievous on the whole, because people rely upon it entirely to clean the teeth, whereas it was always necessary to determine first what was necessary and then procure the appropriate thing.

As to the development of the teeth,—when the child gets its temporary teeth, it should use them upon solid food—food that will require exercise—instead of being fed on puddings and soft pultaceous food. If the teeth are not properly used, the jaw will not be properly developed, and the periosteal attachment will not be so good or stiong. The permanent teeth will also be more apt to be regular if the temporary teeth are properly exercised, so that the jaw may be fully developed and expanded.

Dr. Jay thought that if the dental profession instead of publishing long winded articles in their journals, would write short articles and publish them in the weekly and daily papers, it might be of some benefit.

Dr. Harriman wanted to see the young men of the association induced to speak by having an experience meeting. The older members were obliged to lead in the discussion, and exhausted the subject so thoroughly that the young men stood back. He thought more people were availing themselves of the teaching of the dentists than formerly, and he thought the dentist could do as much good in his sphere as a minister of the gospel, if he did his duty.

Dr. Ulrey said there was no way to overcome the carelessness of patients in respect to their teeth, except by hammering away at them.

Dr. Taft—I will give Dr. Taylor a text and I want him to preach a sermon from it. What is the proper treatment for the teeth of mothers during the period of gestation and lactation.

Dr. Taylor—I have been silent heretofore because I wanted to hear from some of the younger members. I think the text given me is the starting point, and right there we meet with some of the greatest difficulties that present themselves. There are so many peculiar physical conditions of the system that what is good treatment for one case may not be good treatment for another. I have often asked myself what I would do to obtain a good denture under certain conditions if the control of the patient was given me from the beginning of gestation, because I would not undertake the case unless I could control not only the diet but the habits of the mother from beginning onward. Some persons need only tohave the diet restricted in some way. Suppose I take the case of a young mother of rather lymphatic and phlegmatic temperament, whose teeth have been bad from youth, and the father's teeth are not good.

How shall I secure a good denture to the child? First, it is impossible to rear any structure worth any thing without good material to form the structure from. I could not feed this mother on food utterly deficient in the phosphates for instance, because these are taken from the mother directly to the child, and if you cut off this supply, you have not a good denture, as a necessary consequence. Then I should feed this mother on food rich in the phosphates, such as cracked wheat.

In taking away the bran we take away one-half or two-thirds of the phosphates that the wheat contains. We all know that the teeth are formed before birth, even the permanent teeth to a certain extent being developed. One of the first indications of the deposition of dental structure on the pulp is an appearance of redness, showing that the red corpuscles of the blood are taken directly to the teeth and begin to deposit lime. If we take a patient whose blood is poor and system run down, her pale lips and face indicate that the red blood is almost wanting in her system, in ninety-nine cases out of a hundred you would have a poor denture; if good it is because from extraneous means the child has obtained the material necessary for a good denture after its birth.

In some cases a very little irritation produces terrible results, while the same amount of irritation in another mouth is attended with no bad effects. I have a case now where tartar appeares to accumulate in six weeks. I do wish we could get our patients to appreciate the necessity of perfect cleanliness. As to dentrifices, I would just about as soon expect to give a formula for the cure of typhus or pneumonia, as to give a general dentrifice for my patients. I have been importuned to get up a tooth powder, but I have never been able to do so to my satisfaction. I think it much better to write a prescription according to the needs of my particular patients and send them to the drug store to get it filled. I agree with Dr. Reid about cleaning the teeth; we are all at fault there, I am, I know, and yet I am more particular than I used to be. I go over and take off the tartar first with a scaling instrument, and then polish up the surface of the tooth thoroughly with some kind of powder until they are in a perfectly smooth condition. Charcoal used to be a wonderful thing in dentistry, and I have no doubt at all of the importance of using it under certain conditions. If I have sensitive dentine and the secretions of the mouth are viscid and ropy, I like to use charcoal. If I have a dyspeptic patient I would like to feed him on charcoal. Dr. Keely complained of the charcoal getting around the teeth, beneath the gums, that is just why I use it.

Dr. Rawls.—"Hygiene in relation to the teeth, is the same as hygiene in any other part of the system, viz. The best means of preserving the organs. Children should be governed by the same hygienic rules that govern us in after life, in order to obtain a perfect hygienic development. If the child is weak, of course we must strengthen it the best way we can. The diet is not all that is requisite Mothers generally who are enciente, don't take enough exercise, and don't exercise their children enough after they are born, nor do they give their children food that is hard enough, or of sufficien strength of texture, to resist the action of the teeth and develop them. They don't allow the child a sufficient amount of pure air within the room in which it lives. Local means can not be attended to so well in first, as in second dentition.

You will remember that last year the question of how to prevent decay of the teeth around the margins of the gums, where the teeth are deformed, and more especially the bicuspid teeth having large crowns and small necks which favors more rapid decay, was thoroughly discussed; and it seems to me that since then I have got some new ideas that have helped me very materially on this subject. These are the most difficult cases we have anything to do with, for if the decay takes place and has not extended clear through, it will not form a suitable aperture for filling. Another reason is, that the structure there does not prohibit the increase of secretions from below the margins of the gums. You can't save teeth of that kind by filling, but you can save them by instructing your patients to rub them with napkins, cloths or sticks, and you can take your finishing engine and cut away these softened parts. Don't be afraid to do this—they are of no service there whatever. It does no good to leave any portion that has lost its vitality.

Cited the case of a lady whose gums were absorbed probably a sixteenth of an inch below the original connection with the teeth, who by the above treatment had restored the jaw and gums, and whole condition of the mouth to perfect health.

Dr. H. A. Smith.—The difficulty about this subject is, that we don't know anything about the affections accompanying first dentition, practically, except in our own families. Generally if anything is the matter with a child, diarrhœa or anything else, it is attributed to the teeth, whereas the fault usually is with the food. If that is all right, it is time enough then to attribute it to the teeth. There is a considerable prejudice to cutting gums, but I dont know why. They say there is a cicatrix formed which prevents a proper eruption of the teeth. That is not true; the cicatrix is more easily absorbed than the original tissue.

Dr. Roudebush thought it but little use to lance gums until dentition was pretty far advanced. Cited the case of a little boy brought to him with an eruption on the right cheek, for which he prescribed the use of zinc ointment, telling the parents, however, that the child would experience no permanent relief until the tooth made its appearance. He afterward recommended calomel to be sprinkled on it, which dried it up completely, but the child broke out almost immediately, about the rectum, just as it was on the face.

Dr. Smith—What is the reason the child does not erupt teeth without any constitutional disturbance?

Dr. Jameson—They probably would if we had another Adam and Eve to start with.

Dr. C. S. Smith of Ill.—I don't think the question proposed by Dr. Taft can be answered. The causes reach back to the beginning of our civilization and are out of our reach. I believe much of the want of proper development of the teeth of the present generation, is due to the want of proper nourishment. For years we have been taking out of the flour the very materials needed for the building up of the teeth, and nothing has been furnished to supply the want. I don't think it the want of exercise, though I do not underestimate the importance of that, want of exercise will not account for the eruptions of four large teeth in the jaw of a child, not large enough to receive them. This difficulty has been attributed to the mingling of races and that may be true. I am not prepared to say what the cause is, but one thing is certain; we can't make teeth out of starch. Children whose teeth are defective, generally live on soft pultaceous food. I see no way of evercoming this difficulty, unless we can discover some means of neutralising the effects of our civilization, or else relapsing into barbarism. Professor Weatherby, of England, has stated that this trouble is to be largely attributed to the use of iron by the mothers in any form.

Dr. Jay thought tobacco a fruitful cause of trouble by means of its action on the nervous system. If he had his way he would burn the last ounce of tobacco in the world, and destroy the last seed. Those who live five hundred years hence, will see this nation a greater nation of invalids than it is today on that account.

Dr. Harriman thought tobacco acted upon the teeth through the nervous system.

Dr. H. A. Smith thought tobacco and alcohol not so injurious as supposed. As to the effect of food he related the case of a lady who brought her children to him and told him that one of them had been fed on the kind of food he had advised, and requested him to decide which had the best teeth. He pointed out the child with the poorest set of teeth, when the lady said, "Now you are a pretty kind of dentist. He is the only one of my children who has been brought up strictly on your system." It was also the same way in his own family, so that the brown bread theory has failed also. He thought the only hope for the race was on the principle of evolution, or the survival of the fittest. Those having the best teeth would live the longest and eventually the race would be improved.

Dr. Leslie—Every gentleman in the room is more or less acquainted with the influences of the phosphates on the teeth. He had found that the higher classes in Europe who live on the best and richest food generally have the finest sets of teeth, while those people who live on poor food have poor teeth. The phosphates abounded in the rich food, while they were not so plentiful in the poor food; hence the difference. The Scotch people who generally have very good teeth, consume a great deal of oatmeal, but they also consume more whisky per head than any other nation on the face of the earth, and they also use a great deal of tobacco in the form of snuff.

Adjourned until Thursday morning at nine o'clock a. m.

THURSDAY MORNING.

The association was called to order at ten a. m., and the subject of Dental Medication; Local and General, was discussed by the members as follows:

Dr. Hunter said there was one turn he wanted to see this discussion take, and that was with regard to the systemic remedies used. It was called to his mind from the fact of having operated for a young lady who remarked at the time that she thought she was going to have trouble with the tooth, and about a week afterwards came back, and said that it had been sore, and that her physician had given her a remedy that had effectually cured it. I thought it a little peculiar, and I suppose she noticed the smile of incredulity, and said: "Oh, you are an old school man, and don't believe in homœopathic remedies." Her physician told me he had given her chamomilla, and had used it on her before, and found it a very happy remedy. We know that homœopathists use mercurius vivus for the tooth ache, but this was a new remedy for me. I asked him how it acted, and he very freely acknowledged that he did not know, but said that he had used silecia, pulsatilla, chamomilla and mercurius. Now these are remedies we don't know anything about, and if there is anything in them, I think we ought to know it, so I would like to hear from some of the homœopathists of this association.

Dr. Corson said that a prominent homœopathic physician had told him that the remedies would succeed sometimes, and sometimes would not. Frequently local treatment had to be resorted to in the end.

Dr. Taft—We can not to-day hope to do more than have some discussion on a few of the remedies that are available to us.

Medication involves diagnosis and a knowledge of conditions that attach to the part to begin with, or rather that is not the beginning, but it is antecedent to any treatment. A knowledge of true physiology precedes a knowledge of pathology or imperfect physiology. A knowledge of the structure both general and minute, is a necessary attainment. Anatomy is the beginning. General anatomy, minute anatomy, a knowledge of the tissues of their histological character and of their relations, is requisite in the administration of medicines.

Dr. Hunter has just said that the physician with whom he conversed on the subject, frankly confessed he did not know how the cure was affected. He gave the medicine, but did not know why. Are we to rest satisfied with that kind of knowledge? I think any one would be very easily satisfied who would. If you were very sick, Mr. President, you wouldn't want any physician to come in and say, "I have a package of medicines here; I don't know what the effect will be, but I will try them on you and see how they work." Or, if he should say, "I have a medicine here that cured John Smith; I don't know whether your case is at all like his or not, but I think I will try that on you." Or, if he should say, "I have a medicine here that cured several persons, while several others to whom I administered it, died under the treatment. I don't know whether it is just what you want or not, but I think I will try it on you." You wouldn't feel very comfortable under such treatment. But if a man comes to you and says certain conditions exist, which he well understands; and sets about to accomplish a certain object in a certain way, you feel very differently. The idea that one medicine will meet all cases is simply preposterous. Will local treatment meet the difficulty when there is a low grade of vitality? You must resort then to general treatment in connection with local. If you desire an escharotic effect, select the best agent for the purpose. The great trouble is a want of knowledge of general principles. It is difficult to give a general formula and follow it in all cases. That is the real difficulty of the profession. The question should be, what ought to be done in this particular case in hand. He had made experiments with mercurius vivus, and found it to work well sometimes, and to fail frequently.

Dr. Rice—So far as the application of homœopathic remedies is concerned, they follow closely and exactly with as much precision as possible, an accurate diagnosis of the case. That is the theory, but whether the practice bears out the theory is for a man to say who has had some experience. The kind of inflammation for which mercurius vivus is given, is the same kind that is produced by mercurius vivus. They follow out their motto of '*Similia Similibus Curantur.*' Of course, every one will leave entirely out of the question, inflammation caused by decomposing pulp or gases. It would be mere folly to administer remedies in that case. Inflammation will very often pass away of itself, and I think it often does so when mercurius is given and supposed to operate favorably, but it is not fair to the homœopathic profession, to say that mercurius is the only remedy. Dr. Hunter has mentioned others which they claim; but so far as the internal administration of medicine is concerned, I think dentists ought to leave that alone. I think the more dentists attempt to hinge on to the medical profession, and assume the duties of the physician, the less it will benefit him or his patients. And it is an intimation that medical men don't understand these subjects, but medical men do know something about the teeth. The subject is taught in medical colleges and the student who has paid proper attention to the lectures is informed upon it. Of course it is not supposed that he is informed as to operative dentistry, but I think it an insult to medical men to attempt to prescribe for the administration of internal remedies, and whenever the dentist tries that, he is sure to get into trouble.

Dr. Taft—I am very much surprised at Dr. Rice, and sorry he has said what he has, for if it influences any one at all, it will have a tendency to influence them in the wrong direction. How any man can treat an abscess systemically, I don't understand. It seems to me Dr. Rice has ignored that principle entirely, or else don't understand it right. The idea that he advances, which would place a dentist in the position of a mere tinkerer in the mouth is a matter of the greatest surprise. The truth is, and the medical men will not deny it, that many conditions arising from diseased teeth are better understood by the dentist than by the physicians. I have sent patients to physicians for an opinion, and they have sent them back again without giving it.

Now as to '*Similia Similibus Curantur*,' it seems to me absurd. If that were true, the proper remedy for periostitis would be an application of decomposed tooth pulp to the part, for that, more than any other one thing produces periostitis.

A very important subject is exudation; what medication can be employed to properly control and modify this process. This is an important subject. This is a subject with which we have to do in the treatment of exposed pulps, abscesses, etc. It is worth our attention and earnest study, and it is a subject which requires for its clear apprehension, correct knowledge and experience.

Dr. Rice—Dr. Taft expressed himself surprised at me, but I am even more surprised and astonished at him because I regarded him as rather favorable to homœopathy.

Dr. H. A. Smith—I would like to say a word upon the value of rest as a therapeutical treatment of disease. A young man told me this morning that having suffered from indigestion for a long time, his physician put him first upon a diet of milk; three spoonfuls of milk three times a day, thus resting the digesting organs. Cited the case where a tooth had been irritated from tartar, until loss of tissue about it resulted. He attempted to remove it by dilute sulphuric acid and other agents, but could do nothing with it. Then noticed that a false tooth was pressing upon it; removed that pressure, and the tooth is now in a fair way to be restored. Here it was simply rest that the tooth needed. In replanting perfect rest must be given if any beneficial effects are to result. It is not medication; it is rest that cures disease. Not long ago I was struck by an article upon the treatment of consumption, in which rest instead of exercise was advocated.

In reference to homœopathy, Dr. Geo. Watt said there can be but one school of medicine, and when the Vice-President of the Homœopathic Association of Great Britain says that the motto 'like cures like' is a fraud, it seems that Dr. Watt might possibly be right. I think homœopathic doctors now recognize the fact that they can not confine themselves strictly to their own theories. They don't admit this publicly but it is the fact. On the other hand almost all works on materia medica have adopted some of the homœopathic remedies. For instance, Dr. Bartholow's new work mentions aconite. Much of the apparent success of homœopathic remedies is due to the fact that no medication at all is necessary. A distinguished homœopathic physician told me that in a large proportion of cases no medication at all was necessary and hence little pills were just as good as anything else. They could do no harm at least.

Dr. Reid thought Dr. Smith had made the strongest argument in favor of homœopathy that he had yet heard. He advocated rest and that is just what the homœopath does—he don't disturb you at all. He thought physicians and dentists should go hand in hand.

Dr. Sage wanted to say a word in defense of homœopathy. While he was not prepared to announce himself as an advocate of that school, he was convinced that there was much in it that was valuable, and he was not satisfied to sit quietly and hear these slurs cast upon it. Inasmuch as we are discussing the subject of general medication, I think it involves the principles of different schools, and I see no reason why the homœopathic school should not be considered. I am convinced that the general principles of homœopathy are correct, but I am not prepared to adopt everything in that school. I agree with Dr. Taft that the dentist should be able to administer internal remedies. and he ought to take a course of study in order to fit himself for that purpose. If he does not do that he had better leave the matter alone as Dr. Rice said. I think it is presumptuous for an ordinary dentist to attempt to administer constitutional treatment.

But that does not do away with the necessity of preparing ourselves to administer internal remedies. I have sent patients to doctors in this city who seemed to have no adequate conception of what was needed; that too in the case of some of the most prominent physicians, two of whom at least, I could name. I am inclined to think however that medical men are far better able if well educated, to administer medicines than any dentist is. I am also inclined to think that a careful study of the homœopathic school would convince us, that there is not so much absurdity in *Similia Similibus Curantur* as we suppose at first thought. As to the Vice-President of the Homœopathic Association of Great Britain who was mentioned by Dr. Smith, I have been informed that he has been an apostate from the first, and that he has never fully identified himself with the school.

Dr. Taylor—We know that dentists are not generally prepared to meet these cases, but if we are not to bring our profession up to the ability to prescribe for the administration of medicines, why do we teach anatomy and physiology in our schools—why do we have schools at all? I confess that I could not practice operative dentistry at all, unless I could go back to the first principles. You might as well say that eye doctors need not study general principles at all, because they operate upon the eye. Only a few days ago a gentleman came into my office, and said that he had not been out of his house for two weeks. The whole trouble arose from an abscess in one of his molars, which a dentist could have relieved in ten minutes. The physician treated him carefully and well, and he was an excellent physician; just as good as we have in Cincinnati, but this was the mistake he made, and nine out of ten physicians will make first the same mistake, while no intelligent dentist would be excused for making such blunders." Cited the case of a young lady who lost three or four teeth, one after the other, because they were paining her, and she thought they should be removed, whereas it was simply a case of fever and ague, which instead of breaking out in the form of chills, produced this sensation in the teeth. I think it practically impossible for a dentist to do himself, the profession and the public justice unless he is able to meet these conditions.

Dr. Taft—I fully indorse all of Dr. Taylor's statements, they certainly embody the principles of true practice.

Dr. Rice says that periostitis is caused by mechanical irritation, such as gas or fluids confined in the tooth. That is not the case; the great trouble is on account of the poisonous debris there lodged. I am sorry Dr. Rice makes these mistakes; I think he does not do himself justice.

Dr. Smith told us about rest, and I hoped he was going to tell us how rest accomplished the object. Now rest is especially important where there is to be any reproduction, because the plasm thrown out must be free from disturbance. That is the occasion for the rest.

Dr. Taylor—I intended to make a remark or two while Dr. Rice was present. I don't think he is willing to carry out the full force of his remarks. I would like to ask him under what conditions he would give a cathartic. There are conditions where cathartics would be indicated, and there are conditions where an emetic would be better. There are cases where total depletion, such as leaching and lancing the gums would relieve the increased flow of blood to that part, and then we have exudation, as Dr. Taft said.

Dr. Smith—I would give cathartics during stasis, to relieve the stasis, and promote the circulation. To excite the urinary and other organs and carry out excretory matters in the most fluid condition will rapidly restore the normal process of assimilative nutrition.

Dr. Taylor—Why not in determination?

Dr. Smith—That might possibly be overcome in some other way. I think stasis is the indication.

Dr. Taft—I don't think stasis would be relieved by cathartics alone, or any similar medication. When the case has arrived at stasis something that will give increased energy is required. Purgatives will sometimes answer for stasis, but all the way down until you come to stasis, they will invariably operate beneficially. I had a case of abscess from the root of an inferior tooth, which had been discharging from a fistulous opening in the cheek. For a year this case had been in the hands of a prominent physician of this city, without any benefit. This root was taken out, and within a few days afterward this fistulous opening closed, and in a few months afterward the indentation had all disappeared. That is one case, and I could give you many of a similar kind that I have known during the last twenty years. That is nothing against the physician, except that those things about which they know nothing they should let alone. But the presumption generally is that because a man has studied medicine he knows everything; that is not true; no man can in these days know everything.

Dr. Harriman said that if life was spared to him he intended to take a college course in medicine, so that he need not be afraid to undertake any case that may arise. It would take time to accomplish this, but he believed it the duty of the dentist to do so. It is the ambition of the modern dentist to have a medical education, and it is a laudable ambition and should be followed up with the actual acquirement of the object. He believed the allopathic education was the one for dentists. But dentists and physicians must co-operate. Physicians had brought cases to him which seemed to them very complex, but which were really very simple

Dr. Jay thought Dr. Smith struck the keynote, in many cases of alveolar trouble when he spoke of rest. But there is one point of treatment which has not been touched upon since

I came in, and that is sedatives. Not long ago a man came to my office with a crown of the right inferior second molar broken off, while the fangs still remained solid and firm. I gave him ten grains of quinine and one grain of morphine, and told him to take a dose every four hours, and I saw him the next dav and he said the tooth was perfectly well. I don't know whether it was the medicine or the rest. I have used quinine and morphine with good success, though I confess I don't know why it is. It may be that it has a tendency to allay fever and the inflammation then subsides, but I don't attempt to explain it.

Dr. Taft—I suppose the object of the morphine is to relieve pain. Now how is pain produced? By some interruption in the function of the nerve as in neuralgia. If there is swelling, the pressure on the nerve causes pain. If there is poisonous material in the system that may interrupt the proper action of the nerves, and there must be something to neutralize that influence at once. Quinine does that in many instances. Now what is the action of the morphine? They act jointly upon the circulation modifying it so that this pressure is relieved upon the part. Another mode of action is by the obtunding influence upon the nerve tissue, because that can be deprived entirely of its sensibility by these agents; if persisted in too long they destroy the function of the tissue, causing death in that part, so great is their influence over it. Subject passed.

